# Anxiety and depression in dry eye patients during the COVID-19 pandemic: Mental state investigation and influencing factor analysis

**DOI:** 10.3389/fpubh.2022.929909

**Published:** 2022-07-29

**Authors:** Zhuo Chen, Qing He, Qianhui Shi, Yifan Xu, Haibo Yang, Ruihua Wei

**Affiliations:** ^1^Tianjin Key Laboratory of Retinal Functions and Diseases, Tianjin Branch of National Clinical Research Center for Ocular Disease, Eye Institute and School of Optometry, Tianjin Medical University Eye Hospital, Tianjin, China; ^2^Tianjin Social Science Laboratory of Students' Mental Development and Learning, Academy of Psychology and Behavior, Tianjin Normal University, Tianjin, China

**Keywords:** dry eye, anxiety, depression, health anxiety, sleep, COVID-19, linear regression

## Abstract

**Objective:**

Investigate the anxiety and depression states among dry eye (DE) patients during the COVID-19 outbreak and analyze their influence factors.

**Methods:**

The study was conducted in a tertiary eye hospital in Tianjin, China from March–April 2021. Four hundred twenty-eight DE patients were tested with the Ocular Surface Disease Index, Short Healthy Anxiety Inventory, Hospital Anxiety and Depression Scale, and Pittsburgh Sleep Quality Index. Descriptive statistics was used to assess the difference between DE with depression or anxiety among different groups. And multiple linear regression was used to explore factors that influence anxiety and depression in DE patients.

**Results:**

The incidence rates of anxiety and depression among DE patients during COVID-19 were 27.34 and 26.87%, respectively. The proportion with comorbid anxiety and depression was 24.30%. Patients' education level (*t* = −3.001, *P* < 0.05; *t* = −3.631, *P* < 0.05), course of disease (*t* = 2.341, *P* < 0.05; *t* = 2.444, *P* < 0.05), health anxiety (*t* = 3.015, *P* < 0.05; *t* = 2.731, *P* < 0.05), and subjective sleep quality (*t* = 3.610, *P* < 0.05; *t* = 4.203, *P* < 0.05) had certain influences on anxiety and depression.

**Conclusion:**

The results showed that subjective symptoms of DE patients were related to depression and anxiety. Higher education, shorter disease duration, lower health anxiety levels, and better subjective sleep quality were associated with the reduced depressive and anxiety symptoms in DE patients. These findings could be deemed beneficial to the treatment and prevention of DE during the COVID-19 epidemic.

## Introduction

At the end of 2019, the novel coronavirus disease (COVID-19) pandemic started in Wuhan, China. COVID-19 is caused by Severe Acute Respiratory Syndrome (SARS) Coronavirus-2 (CoV-2), characterized by strong infectivity, a long incubation period, and a high fatality rate (1). The outbreak has also caused psychological distress, fear, and had an impact on the coping strategies of community members (2). Moreover, the incidence of depressive, anxiety, and comorbid depression and anxiety symptoms increased significantly during the pandemic, and these negative psychological outcomes may reduce quality of life (3–5). Still, with much about COVID-19 still unknown, healthcare professionals were forced to quickly adapt and learn to mitigate the rapidly rising infection and mortality rates (6). The most effective public health measure to manage the COVID-19 pandemic currently available remains the stay-at-home quarantine (7).

During the quarantine, the increase in work from home and online classes has led to a higher use of visual display terminals (VDTs). Blink intervals during prolonged staring and excessive tear evaporation from electronic screens are significant causes of dry eye (DE) (8). DE is a multifactorial ocular surface disease characterized by an imbalance in tear film homeostasis and associated symptoms, such as ocular surface inflammation, damage, and neurosensory abnormalities (9). Its prevalence ranges from 5–50% (10). The symptoms such as pain, dryness, itching, redness, foreign body sensation, and sensitivity can significantly affect an individual's ability to perform daily tasks, thereby affecting productivity (11, 12). In addition, the economic burden of DE-related productivity loss is becoming more apparent, with research showing that the symptoms of DE cost an estimated $11,302 per person, as well as that the indirect costs account for the largest proportion of these total costs (13).

Simultaneously, numerous studies have found a significantly increased prevalence of depression and anxiety in DE patients (14). Research by van der Vaart et al. (15) revealed an association between DE, depression, and anxiety in more than 40,000 outpatients over the age of 18. Lendrem et al. (16) assessed the mental status of 639 patients with Sjögren's syndrome, finding that patients with anxiety or depressive symptoms accounted for 49.4% of the observed population. Further, Ayaki et al. (17) surveyed 730 people using the Hospital Anxiety and Depression Scale (HADS) and showed that DE patients scored significantly higher than non-DE patients.

In addition, studies have shown that the subjective symptoms of DE patients do not match the severity of objective examination of their ocular surface during clinical treatment (11, 18), nevertheless further findings on this issue remain to be validated. Regarding anxiety and depression in DE patients, the current study describes them as being mainly related to the following factors: sleep disorders, foreign body sensation, long-term chronic pain, decreased visual quality, and various eye discomforts (19). Additionally, social factors and medical expenses can cause anxiety and depression in DE patients (20). Fortunately, as the medical model transitions from biomedical to biopsychosocial, ophthalmologists have recently started paying increased attention to the psychological status of DE patients (21).

The sleep characteristics in DE patients include poor subjective sleep quality, short sleep duration, and prolonged sleep latency (22). The severity of DE symptoms was significantly associated with clinical insomnia (23). Sleep disturbance may cause ocular surface hyperosmolarity to induce an inflammatory response that further damages the tear film, reducing its stability and increasing with tear evaporation, ultimately leading to DE (24, 25). Simultaneously, ocular discomfort and chronic pain from DE can also aggravate sleep disturbance in DE patients (17), and even cause or exacerbate symptoms of mood disorders in patients (26). Negative emotions can further enhance the body's perception of pain and stimulation. This may be due to the psychological modulation of pain perception involving effects on mood and hypnosis (27).

Health anxiety is an exaggerated level of concern about wellness caused by an individual's misinterpretation of somatosensory feelings or physical changes (28). This anxiety distorts the patient's cognition and assessment of their physical condition, resulting in depression, anxiety, other psychological problems, functional impairment, and iatrogenic injury (29). Since the concept of health anxiety was proposed, research on the topic has focused on the neurology, cardiovascular, and endocrinology fields, on samples of middle-aged and older people, and on factors related to their concerns (30, 31). However, few studies (32) have been done on chronic ophthalmic diseases, such as DE (33). Research on health anxiety among DE patients could improve the societal and familial awareness of their psychological condition.

Many recent studies (34–36) have investigated anxiety and depression in DE patients. However, knowledge remains lacking on the influencing factors of these two psychological variables among DE patients. This study aimed to determine the prevalence of Anxiety and Depression and the risk and protective factors that contribute to psychological problems among DE patients. Our findings may help government agencies and ophthalmologists protect the mental health of DE patients against the backdrop of the spread of COVID-19 in China and worldwide.

## Materials and methods

This cross-sectional study was conducted in a tertiary eye hospital in Tianjin, China from March–April 2021. Our pre-study showed that sleep was related to anxiety and depression, the linear regression coefficients (37) were ~0.9 and 2.5, and the standard deviations of sleep, anxiety and depression were 0.8, 4.5, and 4.2, respectively. Assuming that the type I error α of the hypothesis test is 0.05, the type II error β is 0.1, and the sample size is calculated as *n*_anxiety_ = 402, *n*_depression_ = 38, taking the larger value, the sample size is n = 402. Finally, 431 DE patients were enrolled by random sampling. All the patients were treated in the hospital's clinic and were diagnosed by professional ophthalmologists. The inclusion criteria were as follows: (1) diagnosed with DE; (2) 18 years or older, signing the informed consent forms, and voluntarily participating in this study; and (3) clear state of consciousness, thus being able to understand and complete the questionnaire independently. The exclusion criteria were as follows: (1) active disease of the anterior segment; (2) ocular chemical or thermal burns and ocular trauma; (3) a history of eye surgery in the past 3 months; (4) severe mental illness in the past 12 months (38): Schizophrenia (SZ), Bipolar Disorder (BD) and Major Depressive Disorder (MDD), obtained through collecting the psychiatric/past history of patients; (5) a history of organic brain disease, alcohol, or drug dependence; (6) if female, pregnant or lactating; and (7) other severe illnesses or conditions (e.g., unable to respond to the questionnaire, unable to take care of self, or severely illiterate). The study followed the tenets of the Declaration of Helsinki (39). This survey was reviewed and approved by the Medical Ethics Committee of Tianjin Medical University Eye Hospital [2020KY(L)-53]. Participants provided written informed consent to participate in this study and were able to withdraw from the study at any stage during the process. The data was desensitized and cannot be linked back to identify any participants.

### Measures

#### Evaluation of DE

We performed clinical DE tests using first non-invasive tear film break-up time (F-NITBUT) and the Ocular Surface Disease Index (OSDI) scale according to the guidelines recommended by TFOS DEWS II (9). The OSDI scale comprises three questions: ocular symptoms, visual-related functional effects, and environmental triggers. Scores for this scale are divided into three levels based on symptom severity and frequency: with or without (0–12), mild (13–22), moderate (23–32), and severe DE (33–100). The scale is widely used in the clinical evaluation of DE and has good sensitivity and specificity (40).

Tests were performed using the Keratograph 5M to assess objective indicators of DE. The examination was done by the same physician. All patients were examined in the same examination room, in a dark room environment, and with consistent temperature and humidity (41). The abnormal F-NITBUT was defined as <10 s, the abnormal score for OSDI was defined as ≥13, and subjects meeting these two criteria were considered as DE patients (42).

#### Assessment of health anxiety

The Short Healthy Anxiety Inventory (SHAI) was developed by Salkovskis and is widely used to measure the level of health anxiety (43). The scale comprises 18 items related to the likelihood of disease (Illness Likelihood, IL) and the negative outcome of disease (Negative Consequences, NC). Each item features four declarative sentences representing different degrees and is responded on a scale ranging from 0–3 (0, low; 1, mild; 2, moderate; and 3, severe). The total score ranges from 0–54, with higher scores indicating higher anxiety levels. The threshold score for the screening of health anxiety based on the Chinese version of the SHAI is 15 points (44). The Cronbach's alpha for the scale in this study was 0.61.

#### Assessment of anxiety and depression

Zigmond and Snaith (45) created the HADS in 1983. It is mainly used to screen anxiety and depression in general hospital patients. The scale comprises 14 items, with seven assessing depression and seven measuring anxiety. Items are responded on a 4-point scale (0–3 points). If the total score for anxiety and depression is greater than or equal to 8, it indicates that these conditions are present. The Chinese version of the scale has good reliability and validity (46). The Cronbach's alpha for the scale in this study was 0.80.

#### Sleep quality assessment

The Pittsburgh Sleep Quality Index (PSQI) was compiled by Buysse, a psychiatrist at the University of Pittsburgh, in 1989 (47). The Chinese version of the PSQI has demonstrated good internal consistency and construct validity (48). The PSQI is used to assess participants' sleep quality over the last month. Its 18 items consist of seven components, including sleep quality, time taken to fall asleep, time to sleep, sleep efficiency and disorders, hypnotic drugs, and daytime dysfunction. Each component is scored on a scale of 0–3, and the cumulative score of each component comprises the total PSQI score. The total score ranges from 0–21. The higher the score, the worse the sleep quality. It is used for clinical and basic research on sleep quality evaluation. A score exceeding 6 indicates the presence of a sleep disorder (48). The Cronbach's alpha for the scale in this study was 0.67.

### Statistical analysis

The questionnaires were entered and processed using the commercial software SPSS, of version 23.0. Descriptive statistics, including frequency, percentage, normality, mean, *t*-test, and the χ^2^ test were used to assess the difference between DE with depression or anxiety among different groups. Additionally, univariate regression and multiple linear regression analyses were performed to investigate which variables could influence the development of other variables. *P* < 0.05 was set as the significance level.

## Results

Among the 431 DE patients enrolled, 428 (99.30%) completed the questionnaire and met the inclusion criteria, with an average age of 48.20 ± 15.09 years. Table 1 presents the descriptive statistics of the primary study variables. We observed similar proportions of DE patients with anxiety (117 of 428, 27.34%) and depressive symptoms (115, 26.87%) in the sample. The proportion of DE patients with comorbid anxiety and depression was 24.30% (104), indicating that most anxiety or depression patients were of this type. Based on a 6-point cutoff, 65.40% (280) patients had sleep disorder, and the mean total score of each dimension is presented in Table 2. The mean total scores for the OSDI, F-NITBUT, and health anxiety were 44.41 ± 15.99, 5.34 ± 2.30, and 16.14 ± 4.83, respectively (Table 2).

**Table 1:** Demographic comparison of influencing factors of depression and anxiety in DE patients.

**Group**	**Depression (*****n*** = **115)**	**NO Depression (*****n*** = **313)**	χ^2^	***P***	**Anxiety (*****n*** = **117)**	**NO Anxiety (*****n*** = **311)**	χ^2^	***P***
Age^t^	50.76 ± 14.70	47.26 ± 15.14	−2.133	0.034	50.74 ± 14.98	47.25 ± 15.05	−2.140	0.033
Sex			0.003	0.959			1.964	0.161
Male	32 (26.67%)	88 (73.33%)			27 (22.50%)	93 (77.50%)		
Female	83 (26.95%)	225 (73.05%)			90 (29.22%)	218 (70.78%)		
Menstruation			3.197	0.074			2.585	0.138
Menopause	49 (31.41%)	107 (68.59%)			52 (33.33%)	104 (66.67%)		
Non menopause	34 (22.37%)	118 (77.63%)			38 (25.00%)	114 (75.00%)		
BMI			2.377	0.498			4.201	0.241
<18.5	3 (14.29%)	18 (85.71%)			2 (9.52%)	19 (90.48%)		
18.5 ≤ BMI <25	74 (28.68%)	184 (71.32%)			76 (29.46%)	182 (70.54%)		
25 ≤ BMI <30	34 (25.95%)	97 (74.05%)			35 (26.72%)	96 (73.28%)		
≥30	4 (22.22%)	14 (77.78%)			4 (22.22%)	14 (77.78%)		
Family status			3.274	0.070		3.542	0.060
Single	9 (16.67%)	45 (83.33%)			9 (16.67%)	45 (83.33%)		
Married	106 (28.27%)	268 (71.73%)			108 (28.88%)	266 (71.12%)		
Household location			7.714	0.005			8.563	0.003
Urban	82 (23.91%)	261 (76.09%)			83 (24.19%)	260 (75.81%)		
countryside	33 (38.82%)	52 (61.18%)			34 (61.82%)	51 (38.18%)		
Monthly Income			7.190	0.005			11.134	0.011
≤ 2,000 CNY	8 (53.33%)	7 (46.67%)			9 (60.00%)	6 (40.00%)		
2,000–5,000 CNY	32 (31.07%)	71 (68.93%)			27 (26.21%)	76 (73.79%)		
5,000–8,000 CNY	39 (25.83%)	112 (74.17%)			46 (30.46%)	105 (69.54%)		
>8,000 CNY	36 (22.64%)	123 (77.36%)			35 (22.01%)	124 (77.99%)		
Education levels			10.933	0.012			10.969	0.012
Primary Education	16 (42.11%)	22 (57.89%)			17 (44.74%)	21 (55.26%)		
Middle School Education	29 (34.94%)	54 (65.06%)			29 (34.94%)	54 (65.06%)		
High School Education	29 (26.36%)	81 (73.64%)			26 (23.64%)	84 (76.36%)		
University or higher	41 (20.82%)	156 (79.18%)			45 (22.84%)	152 (77.16%)		
Course of disease			7.994	0.046			10.284	0.016
≤ 1year	45 (22.72%)	153 (77.28%)			48 (24.24%)	150 (75.76%)		
1–3year	44 (28.03%)	113 (71.97%)			38 (24.20%)	119 (75.80%)		
3–5year	15 (29.42%)	36 (70.58%)			21 (41.48%)	30 (58.52%)		
>5 year	11 (50.00%)	11 (50.00%)			10 (45.45%)	12 (54.55%)		
Frequency of visit (Within 1 year)			5.078	0.166			7.659	0.054
First visit	47 (23.86%)	150 (76.14%)			44 (22.34%)	153 (77.66%)		
≤ 6 times	50 (26.88%)	136 (73.12%)			54 (29.03%)	132 (70.97%)		
6–12 times	13 (38.24%)	21 (61.76%)			15 (44.12%)	19 (55.88%)		
>12 times	5 (45.45%)	6 (54.55%)			4 (36.36%)	7 (63.64%)		

**Table 2:** Descriptive statistics for sleep condition and health anxiety in DE patients.

**Variable**	**Mean**	**SD**
PSQI total score	9.16	4.83
Subjective Sleep Quality	1.31	0.93
Sleep Latency	1.47	1.20
Sleep Duration	1.25	1.07
Sleep Quality	1.36	1.16
Habitual Sleep Efficiency	1.06	0.52
Sleep Disturbance	0.40	0.93
Used Sleep Medication	2.30	2.01
Daytime Dysfunction	1.25	1.07
OSDI	44.41	15.99
F-NITBUT	5.34	2.30
Health anxiety	16.13	4.83

First, univariate regression analysis was used to screen for the influencing factors of anxiety and depression (dependent variables). The independent variables were the same for both analyses, including significant demographic variables (family location, monthly income, education level, disease duration), the score for OSDI, TBUT, health anxiety, and the seven dimensions of the PSQI. The results showed that, without considering other factors, the factors influencing anxiety symptoms in DE patients were demographic variables, score for OSDI, health anxiety, subjective sleep quality, sleep latency, sleep disturbance, sleep medication used, and daytime dysfunction. Depressive symptoms were influenced by demographic variables, score for OSDI, health anxiety, subjective sleep quality, sleep latency, sleep disturbance, and sleep medication used.

Then, multiple stepwise regression analyses were conducted to exclude the influence of confounding factors. All the meaningful variables included in the univariate regression above entered the regression equation, with anxiety or depression as the dependent variable. The statistical results of the four multiple regression models with anxiety and depression as dependent variables are shown in Tables 3, 4 (*P* < 0.01).

**Table 3:** Summary of multiple regression models of influencing factors of anxiety.

**Model**	***R***	*R* ^2^	**Adjusted** *R*^2^	***SE***	**Change statistics**				
					***R2*** **change**	***F*** **change**	***df1***	***df2***	***P***
1	0.179	0.032	0.030	2.95319	0.032	14.040	1	426	0.000
2	0.232	0.054	0.049	2.92296	0.022	9.858	1	425	0.002
3	0.274	0.075	0.068	2.89349	0.021	9.701	1	424	0.002
4	0.295	0.087	0.078	2.87831	0.012	5.482	1	423	0.020

*Model 1 Include independent variables: Subjective Sleep Quality, Model 2 Include independent variables: Subjective Sleep Quality, Education Level, Model 3 Include independent variables: Subjective Sleep Quality, Education Level, Health Anxiety, Model 4 Include independent variables: Subjective Sleep Quality, Education Level, Health Anxiety, Course of Disease*.

**Table 4:** Summary of multiple regression models of influencing factors of depression.

**Model**	***R***	*R* ^2^	**Adjusted** ***R**^2^*	***SE***	**Change statistics**				
					***R2*** **change**	***F*** **change**	***df1***	***df2***	***P***
1	0.236	0.056	0.053	3.07813	0.056	25.071	1	426	0.000
2	0.295	0.087	0.083	3.02982	0.032	14.692	1	425	0.000
3	0.326	0.106	0.100	3.00115	0.019	9.158	1	424	0.003
4	0.348	0.121	0.113	2.97988	0.015	7.076	1	423	0.008

*Model 1 Include independent variables: Subjective Sleep Quality, Model 2 Include independent variables: Subjective Sleep Quality, Education Level, Model 3 Include independent variables: Subjective Sleep Quality, Education Level, Health Anxiety, Model 4 Include independent variables: Subjective Sleep Quality, Education Level, Health Anxiety, Course of Disease*.

The statistical results of all coefficients in the linear regression with anxiety and depression as dependent variables are shown in Tables 5, 6. Specifically, education level (*B* = −0.418, *t* = −3.001, *P* < 0.05), disease duration (*B* = 0.383, *t* = 2.341, *P* < 0.05), health anxiety (*B* = 0.087, *t* = 3.015, *P* < 0.05), and subjective sleep quality (*B* = 0.539, *t* = 3.610, *P* < 0.05) also had a significant effect on anxiety. Meanwhile, education level (*B* = −0.523, *t* = −3.631, *P* < 0.05), disease duration (*B* = 0.415, *t* = 2.444, *P* < 0.05), health anxiety (*B* = 0.082, *t* = 2.731, *P* < 0.05), and subjective sleep quality (*B* = 0.673, *t* = 4.203, *P* < 0.05) had a significant effect on depression.

**Table 5:** Summary of linear regression model coefficients of influencing factors of anxiety.

**Variable**	**Univariate regression**				**Multiple linear regression**			
	***B***	**Beta**	***t***	***P***	***B***	**Beta**	***t***	***P***
Monthly income	−0.357	−0.103	−2.139	0.033				
Education levels	−0.453	−0.151	−3.162	0.002	−0.418	−0.140	−3.001	0.003
Course of disease	0.463	0.132	2.749	0.006	0.383	0.109	2.341	0.020
Health anxiety	0.094	0.151	3.160	0.002	0.087	0.141	3.015	0.030
F-NITBUT	−0.088	0.063	−1.404	0.161				
OSDI	0.026	0.009	2.862	0.004				
SSQ	0.574	0.179	3.747	0.000	0.539	0.168	3.610	0.000
SL	0.275	0.111	2.296	0.022				
SD	0.028	0.135	0.206	0.837				
HES	0.102	0.125	0.813	0.416				
SDE	0.795	0.139	2.889	0.004				
USM	0.480	0.149	3.120	0.002				
DD	0.170	0.114	2.370	0.018				

*OSDI, Ocular Surface Disease Index; TBUT, SSQ, Subjective Sleep Quality; SL, Sleep Latency; SD, Sleep Duration; HSE, Habitual Sleep Efficiency; SDE, Sleep Disturbance, USM, Used Sleep Medication; DD, Daytime Dysfunction*.

**Table 6:** Summary of linear regression model coefficients of influencing factors of depression.

**Variable**	**Univariate regression**				**Multiple Linear Regression**			
	***B***	**Beta**	***t***	***P***	***B***	**Beta**	***t***	***P***
Monthly Income	−0.524	−0.143	−2.991	0.003				
Education levels	−0.588	−0.186	−3.914	0.000	−0.523	−0.166	−3.631	0.000
Course of disease	0.544	0.147	3.067	0.002	0.415	0.112	2.444	0.015
Health anxiety	0.093	0.142	2.971	0.003	0.082	0.125	2.731	0.007
F-NITBUT	−0.051	0.067	−0.761	0.447				
OSDI	0.028	0.009	2.904	0.004				
SSQ	0.799	0.236	5.007	0.000	0.673	0.198	4.203	0.000
SL	0.292	0.111	2.314	0.021				
SD	0.205	0.142	1.442	0.150				
HES	0.253	0.131	1.929	0.054				
SDE	0.874	0.145	3.024	0.003				
USM	0.821	0.183	3.850	0.000				
DD	0.139	0.076	1.833	0.067				

*OSDI, Ocular Surface Disease Index; TBUT, SSQ, Subjective Sleep Quality; SL, Sleep Latency; SD, Sleep Duration; HSE, Habitual Sleep Efficiency; SDE, Sleep Disturbance, USM, Used Sleep Medication; DD, Daytime Dysfunction*.

## Discussion

This study used multiple linear regression to investigate a group of DE patients in outpatient clinics during the COVID-19 pandemic. Research shows that the prevalence of anxiety disorder is ~10% in the general population (49), implying that the rates in the current study (27.24%) were much higher. Moreover, depression rates in the general population are estimated to range between 3.6 and 8.5% (50). Again, the study rate of 26.87% was considerably higher than the range for the general population. One potential reason for the high incidence of anxiety and depression in this study may be that the COVID-19 pandemic and self-isolation measures have influenced the population's mental health. Specifically, research shows that mental health problems such as acute stress, anxiety, and depression are positively associated with the pandemic (51–53). Another possible explanation is that the pandemic has had a negative impact on the way people live, with students and staff forced to study and work online for extended periods of time. When people focus on digital screens, their blink intervals tend to be longer, dropping in frequency from ~18 to 3 or 4 per min (54). Simultaneously, the intensity or strength of the blink is reduced, and partial blinking occurs, resulting in the eyelid not fully covering the corneal surface (55). This increases tear evaporation, which may increase the incidence of DE and worsen DE symptoms (56, 57). Moreover, long-term chronic ocular surface pain, irritation, visual fatigue and other subjective symptoms of DE can negatively impact patients' cognitive processes and mental health (58). However, some studies have shown that depressive symptoms and severity in DE patients are not related to the severity of DE signs or symptoms (59). Confirmatory conclusions require further research in the future. This study also found a comparatively higher proportion of combined anxiety and depression (24.30%), and previous studies have shown that combined depression and anxiety may impair social functioning, reduce quality of life, and be more likely to increase the recurrence of mental illness and lead to suicide (60). Society and health care institutions should pay attention to this (61).

Consistent with our results, multiple studies have found (11, 32, 62) that scores for anxiety and depression scales were not associated with the objective examination of DE. The symptoms of DE can be considered as being subjective, entailing that they are affected by individual differences in sensitivity to DE signs and basic health conditions. For instance, individuals with DE may experience different symptoms even if they have the same objective examination of their ocular surface. Additionally, irritant ocular symptoms may impact visual performance and perception in DE patients (63). Visual perception disturbances, in turn, may affect visual performance and lead to or exacerbate depression and anxiety (64, 65). A study showing the role of health anxiety, depression, and anxiety symptoms in DE may explain the lack of correlation between symptoms and objective signs of disease (32). Although there are few studies on the pathological mechanism of anxiety and depression caused by DE, it has been determined that the high expression of inflammatory cytokines in the central nervous system of patients with Sjögren's syndrome is closely related to the occurrence of depression (66). Indeed, elevated levels of chronic inflammatory cytokines lead to changes in neuroendocrine and central nervous system metabolites, which can then lead to or exacerbate anxiety and depression symptoms (67). Additionally, anti-anxiety/depressant medication is a risk factor for DE (68). However, there are also studies indicate (18, 69) that there exists an association between uncomfortable symptoms and signs of DE, but no firm conclusions can be drawn for the time being.

The standard coefficient of education level was negative. This indicated that the lower the patient's education level, the more likely they were to develop depression and anxiety. This result may reflect that DE patients with lower education levels had less economic and social resources, could not scientifically and rationally manage stressful life events such as the DE, and paid less attention to own health problems. These possible explanations were shown in a study in the Tibetan areas of China (20). Furthermore, prior research shows that patients with lower education levels were more likely to live in disadvantaged, hazardous, or unhealthy occupations, have inadequate nutrition and exercise habits (70), reside in unfavorable environments with poor medical care, and develop depression and anxiety over time (71). A previous study showed that higher education levels are associated with depressive symptoms (72), which needs to be further verified.

The standard coefficient of the course of the disease was positive. This result indicated that with the prolongation of the disease course, the patient's anxiety and depression worsened. During clinical treatment, with the prolongation of the course of the disease, the confidence of DE patients in recovery is likely to be negatively affected. Furthermore, they may become increasingly worried about the severity of DE and the effect of treatment, which may then lead to depression and anxiety. This finding was consistent with previous studies on other diseases, which show that long disease duration, severe symptoms, and impaired social function were associated with anxiety and depression (73).

Among the 428 DE patients in this study, 61.4% showed health anxiety. This number was higher than the prevalence of health anxiety in the general population, which was 5% in one study (74), and 9% in another research on comprehensive medical institutions (31). As shown in Tables 5, 6, health anxiety had a significant impact on anxiety and depression. Based on research on health beliefs and health anxiety (75), we infer that patients with health anxiety may be more sensitive to somatic and/or physical symptoms for some specific reasons (e.g., stress from past unfortunate and negative events) and prone to repetitively seeking out medical consultation and examination. Furthermore, patients with a high health anxiety disorder may be prone to viewing DE as a persistent disorder after experiencing a period of eye discomfort. Health anxiety can lead to poor perception of physical performance and have a significant negative impact on daily life, leading to a gradual shift in the patient's coping style toward negativity. This coping style reduces the patient's recovery expectations. These individuals do not actively cooperate with treatment. Patients will show a sense of hopelessness, which eventually leads to depression and anxiety (76, 77). The commonsense model of self-regulation (78) also posits that personal beliefs about threats (e.g., chronic diseases) can be generated by individuals and affect how they cope with illness. This may cause patients to often fail to follow doctor's orders or take their medicines on time. These behaviors worsen their condition and lead to more severe anxiety and depression. Previous studies have also confirmed that health anxiety will affect individuals' correct cognition and assessment of their physical conditions (79), resulting in psychological problems such as anxiety and depression, potentially leading to functional impairment and iatrogenic injury (29). During the treatment process, the therapist needs to gain the patient's trust, show understanding and sympathy for the patient, and cannot focus too much on very subtle physical symptoms. Still, the patient's physical health cannot be ignored (80).

The mean total score for PSQI was 9.16 ± 4.83, indicating poor subjective sleep quality, which is a significant component of sleep. Lack of sleep can cause lipid metabolism disorders, thereby destroying the microvilli morphology of corneal epithelial cells, so that tears cannot be adsorbed on the cornea's surface. Moreover, decreased sleep quality can disrupt the circadian rhythm of tear osmolarity, leading to ocular surface hyperosmolarity and tear film instability. These conditions are believed to be the main factors causing DE (9, 81, 82). Among DE symptoms, eye discomfort and chronic pain are associated with sleep quality, stress perception, as well as anxiety and depression as (83), with more than 40% of DE patients experiencing poor sleep quality (17, 84). Simultaneously, sleep quality is closely related to anxiety and depression. Yoo et al. (85) have found that sleep deprivation weakened connections between the amygdala, medial prefrontal cortex, and orbitofrontal cortex, and this compromise affects the regulation function of the emotional disturbance network and leads to affective disorders. Gujar et al. (86) also found weakened connections between the medial prefrontal cortex and the orbitofrontal cortex in sleep-deprived patients, disrupting the mesolimbic reward brain network. Another study (87) showed a slight two-way link between depression and insomnia, showing that structural and functional abnormalities of the amygdala, prefrontal cortex, anterior cingulate cortex, and insula may be the underlying causes of insomnia and mood disorders. In conclusion, poor sleep quality is associated with DE, and is more likely to lead to individual metabolic dysfunction and neurotransmitter secretion disorders, cognitive decline, depression and anxiety (88), which should be noted in clinical practice.

Patients can also manage the disease scientifically in daily life to help with the treatment of DE, such as appropriately increasing the environmental humidity, exercising outdoors, wearing protective glasses, and ingesting food that can promote tear secretion. The limitations of this study are as follows. First, the cross-sectional design precludes the possibility of causal analysis. Second, the subjective questionnaire survey method was used to investigate the sleep quality of DE patients, and no objective instruments were used to detect sleep conditions. Subjective reporting may produce distorted and inaccurate participant accounts of sleep time and delay. Third, the patients come from a single region (Tianjin, China, and surrounding areas), so these findings may not apply to other regions or countries because social and cultural factors may also play essential roles in disease formation. Fourth, this study did not assess social support, which may be an important protective factor against depression and anxiety during the COVID-19 pandemic (89, 90).

## Conclusion

Our findings indicated some risk factors for anxiety and depressive symptoms in DE patients and directly inform the development of psychological interventions for DE patients to minimize the psychological impact of the COVID-19 pandemic. This study also provided a research basis for evaluating DE prevention, control, and treatment efforts during the COVID-19 pandemic. In the future, longitudinal studies are warranted and will enable us to systematically understand the process and laws of the psychological development of DE patients.

## Data availability statement

The raw data supporting the conclusions of this article will be made available by the authors, without undue reservation.

## Ethics statement

The studies involving human participants were reviewed and approved by Medical Ethics Committee of Tianjin Medical University Eye Hospital. The patients/participants provided their written informed consent to participate in this study.

## Author contributions

ZC, QH, and QS: material preparation, data collection, and analysis were performed. The first draft of the manuscript was written by ZC and QH. All authors commented on previous versions of the manuscript. All authors contributed to the study conception, design, read, and approved the final manuscript.

## Funding

This work was supported by Grant 82070929 from the National Natural Science Foundation of China and Tianjin Key Medical Discipline (Specialty) Construction Project.

## Conflict of interest

The authors declare that the research was conducted in the absence of any commercial or financial relationships that could be construed as a potential conflict of interest.

## Publisher's note

All claims expressed in this article are solely those of the authors and do not necessarily represent those of their affiliated organizations, or those of the publisher, the editors and the reviewers. Any product that may be evaluated in this article, or claim that may be made by its manufacturer, is not guaranteed or endorsed by the publisher.

## References

[1] Ahn DG Shin HJ Kim MH Lee S Kim HS Myoung J. Current status of epidemiology, diagnosis, therapeutics, and vaccines for Novel Coronavirus Disease 2019 (COVID-19). J Microbiol Biotechnol. (2020) 30:313–24. 10.4014/jmb.2003.03011 32238757 PMC9728410

[2] Bahar Moni AS Abdullah S Bin Abdullah M Kabir MS Alif SM Sultana F. Psychological distress, fear and coping among Malaysians during the COVID-19 pandemic. PLoS One. (2021) 16:e0257304. 10.1371/journal.pone.0257304 34506576 PMC8432783

[3] Leong Bin Abdullah MFI Mansor NS Mohamad MA Teoh SH. Quality of life and associated factors among university students during the COVID-19 pandemic: a cross-sectional study. BMJ Open. (2021) 11:e048446. 10.1136/bmjopen-2020-048446 34620656 PMC8507402

[4] Woon LS Mansor NS Mohamad MA Teoh SH Leong Bin Abdullah MFI. Quality of life and its predictive factors among healthcare workers after the end of a movement lockdown: the salient roles of COVID-19 stressors, psychological experience, and social support. Front Psychol. (2021) 12:652326. 10.3389/fpsyg.2021.652326 33897561 PMC8062802

[5] Leong Bin Abdullah MFI Ahmad Yusof H Mohd Shariff N Hami R Nisman NF Law KS. Depression and anxiety in the Malaysian urban population and their association with demographic characteristics, quality of life, and the emergence of the COVID-19 pandemic. Curr Psychol. (2021) 40:6259–70. 10.1007/s12144-021-01492-2 33623353 PMC7892323

[6] Islam MS Rahman KM Sun Y Qureshi MO Abdi I Chughtai AA. Current knowledge of COVID-19 and infection prevention and control strategies in healthcare settings: a global analysis. Infect Control Hosp Epidemiol. (2020) 41:1196–206. 10.1017/ice.2020.237 32408911 PMC7253768

[7] Pellegrini M Bernabei F Scorcia V Giannaccare G. May home confinement during the COVID-19 outbreak worsen the global burden of myopia? Graefes Arch Clin Exp Ophthalmol. (2020) 258:2069–70. 10.1007/s00417-020-04728-2 32367286 PMC7197632

[8] Uchino M Yokoi N Uchino Y Dogru M Kawashima M Komuro A. Prevalence of dry eye disease and its risk factors in visual display terminal users: the Osaka study. Am J Ophthalmol. (2013) 156:759–66. 10.1016/j.ajo.2013.05.040 23891330

[9] Craig JP Nichols KK Akpek EK Caffery B Dua HS Joo CK. TFOS DEWS II definition and classification report. Ocul Surf. (2017) 15:276–83. 10.1016/j.jtos.2017.05.008 28736335

[10] Farrand KF Fridman M Stillman I Schaumberg DA. Prevalence of diagnosed dry eye disease in the United States among adults aged 18 years and older. Am J Ophthalmol. (2017) 182:90–8. 10.1016/j.ajo.2017.06.033 28705660

[11] Li M Gong L Chapin WJ Zhu M. Assessment of vision-related quality of life in dry eye patients. Invest Ophthalmol Vis Sci. (2012) 53:5722–7. 10.1167/iovs.11-9094 22836767

[12] Hallak JA Jassim S Khanolkar V Jain S. Symptom burden of patients with dry eye disease: a four domain analysis. PLoS ONE. (2013) 8:e82805. 10.1371/journal.pone.0082805 24349365 PMC3862676

[13] McDonald M Patel DA Keith MS Snedecor SJ. Economic and humanistic burden of dry eye disease in Europe, North America, and Asia: a systematic literature review. Ocul Surf. (2016) 14:144–67. 10.1016/j.jtos.2015.11.002 26733111

[14] Wan KH Chen LJ Young AL. Depression and anxiety in dry eye disease: a systematic review and meta-analysis. Eye. (2016) 30:1558–67. 10.1038/eye.2016.186 27518547 PMC5177754

[15] van der Vaart R Weaver MA Lefebvre C Davis RM. The association between dry eye disease and depression and anxiety in a large population-based study. Am J Ophthalmol. (2015) 159:470–4. 10.1016/j.ajo.2014.11.028 25461298 PMC4329250

[16] Lendrem D Mitchell S McMeekin P Bowman S Price E Pease CT. Health-related utility values of patients with primary Sjögren's syndrome and its predictors. Ann Rheum Dis. (2014) 73:1362–8. 10.1136/annrheumdis-2012-202863 23761688

[17] Ayaki M Kawashima M Negishi K Tsubota K. High prevalence of sleep and mood disorders in dry eye patients: survey of 1,000 eye clinic visitors. Neuropsychiatr Dis Treat. (2015) 11:889–94. 10.2147/NDT.S81515 25848288 PMC4386766

[18] Johnson ME. The association between symptoms of discomfort and signs in dry eye. Ocul Surf. (2009) 7:199–211. 10.1016/S1542-0124(12)70187-8 19948103

[19] Ayaki M Kawashima M Negishi K Kishimoto T Mimura M Tsubota K. Sleep and mood disorders in dry eye disease and allied irritating ocular diseases. Sci Rep. (2016) 6:22480. 10.1038/srep22480 26927330 PMC4772386

[20] Lu P Chen X Liu X Yu L Kang Y Xie Q. Dry eye syndrome in elderly Tibetans at high altitude: a population-based study in China. Cornea. (2008) 27:545–51. 10.1097/ICO.0b013e318165b1b7 18520503

[21] Bitar MS Olson DJ Li M Davis RM. The correlation between dry eyes, anxiety and depression: the sicca, anxiety and depression study. Cornea. (2019) 38:684–9. 10.1097/ICO.0000000000001932 30950896

[22] Lee W Lim SS Won JU Roh J Lee JH Seok H. The association between sleep duration and dry eye syndrome among Korean adults. Sleep Med. (2015) 16:1327–31. 10.1016/j.sleep.2015.06.021 26498231

[23] Galor A Seiden BE Park JJ Feuer WJ McClellan AL Felix ER. The association of dry eye symptom severity and comorbid insomnia in US veterans. Eye Contact Lens. (2018) 44 Suppl 1:S118–S24. 10.1097/ICL.0000000000000349 28181961 PMC5500440

[24] Ayaki M Tsubota K Kawashima M Kishimoto T Mimura M Negishi K. Sleep disorders are a prevalent and serious comorbidity in dry eye. Invest Ophthalmol Vis Sci. (2018) 59:Des143-Des50. 10.1167/iovs.17-23467 30481819

[25] Kojima T Dogru M Kawashima M Nakamura S Tsubota K. Advances in the diagnosis and treatment of dry eye. Prog Retin Eye Res. (2020) 78:100842. 10.1016/j.preteyeres.2020.100842 32004729

[26] Ohayon MM Schatzberg AF. Using chronic pain to predict depressive morbidity in the general population. Arch Gen Psychiatry. (2003) 60:39–47. 10.1001/archpsyc.60.1.39 12511171

[27] Apkarian AV Bushnell MC Treede RD Zubieta JK. Human brain mechanisms of pain perception and regulation in health and disease. Eur J Pain. (2005) 9:463–84. 10.1016/j.ejpain.2004.11.001 15979027

[28] Asmundson GJ Abramowitz JS Richter AA Whedon M. Health anxiety: current perspectives and future directions. Curr Psychiatry Rep. (2010) 12:306–12. 10.1007/s11920-010-0123-9 20549396

[29] Lee S Creed FH Ma YL Leung CM. Somatic symptom burden and health anxiety in the population and their correlates. J Psychosom Res. (2015) 78:71–6. 10.1016/j.jpsychores.2014.11.012 25466323

[30] Bourgault-Fagnou MD Hadjistavropoulos HD. Understanding health anxiety among community dwelling seniors with varying degrees of frailty. Aging Ment Health. (2009) 13:226–37. 10.1080/13607860802380664 19347689

[31] Tyrer P Cooper S Crawford M Dupont S Green J Murphy D. Prevalence of health anxiety problems in medical clinics. J Psychosom Res. (2011) 71:392–4. 10.1016/j.jpsychores.2011.07.004 22118381

[32] Szakáts I Sebestyén M Németh J Birkás E Purebl G. The role of health anxiety and depressive symptoms in dry eye disease. Curr Eye Res. (2016) 41:1044–9. 10.3109/02713683.2015.1088955 26642862

[33] Donthineni PR Shanbhag SS Basu S. An evidence-based strategic approach to prevention and treatment of dry eye disease, a modern global epidemic. Healthcare (Basel). (2021) 9:89. 10.3390/healthcare9010089 33477386 PMC7830429

[34] Hallak JA Tibrewal S Jain S. Depressive symptoms in patients with dry eye disease: a case-control study using the beck depression inventory. Cornea. (2015) 34:1545–50. 10.1097/ICO.0000000000000641 26426334 PMC4636920

[35] Na KS Han K Park YG Na C Joo CK. Depression, stress, quality of life, and dry eye disease in Korean women: a population-based study. Cornea. (2015) 34:733–8. 10.1097/ICO.0000000000000464 26002151

[36] Zhang Y Lin T Jiang A Zhao N Gong L. Vision-related quality of life and psychological status in Chinese women with Sjogren's syndrome dry eye: a case-control study. BMC Womens Health. (2016) 16:75. 10.1186/s12905-016-0353-z 27955668 PMC5154065

[37] Kutner MH Nachtsheim CJ Neter J. Applied Linear Regression Models. 4th ed. Chicago, IL: McGraw-Hill (2004).

[38] Salzer MS Brusilovskiy E Townley G. National estimates of recovery-remission from serious mental illness. Psychiatr Serv. (2018) 69:523–8. 10.1176/appi.ps.201700401 29385961

[39] World Medical Association. World Medical Association Declaration of Helsinki: ethical principles for medical research involving human subjects. Jama. (2013) 310:2191–4. 10.1001/jama.2013.281053 24141714

[40] Vitali C Moutsopoulos HM Bombardieri S. The European Community Study Group on diagnostic criteria for Sjögren's syndrome. Sensitivity and specificity of tests for ocular and oral involvement in Sjögren's syndrome. Ann Rheum Dis. (1994) 53:637–47. 10.1136/ard.53.10.637 7979575 PMC1005429

[41] Guarnieri A Carnero E Bleau AM López de Aguileta Castaño N Llorente Ortega M Moreno-Montañés J. Ocular surface analysis and automatic non-invasive assessment of tear film breakup location, extension and progression in patients with glaucoma. BMC Ophthalmol. (2020) 20:12. 10.1186/s12886-019-1279-7 31906897 PMC6945571

[42] Wolffsohn JS Arita R Chalmers R Djalilian A Dogru M Dumbleton K. TFOS DEWS II diagnostic methodology report. Ocul Surf. (2017) 15:539–74. 10.1016/j.jtos.2017.05.001 28736342

[43] Salkovskis PM Rimes KA Warwick HM Clark DM. The Health Anxiety Inventory: development and validation of scales for the measurement of health anxiety and hypochondriasis. Psychol Med. (2002) 32:843–53. 10.1017/S0033291702005822 12171378

[44] Zhang Y Liu R Li G Mao S Yuan Y. The reliability and validity of a Chinese-version Short Health Anxiety Inventory: an investigation of university students. Neuropsychiatr Dis Treat. (2015) 11:1739–47. 10.2147/NDT.S83501 26213472 PMC4509540

[45] Zigmond AS Snaith RP. The hospital anxiety and depression scale. Acta Psychiatr Scand. (1983) 67:361–70. 10.1111/j.1600-0447.1983.tb09716.x 6880820

[46] Li Q Lin Y Hu C Xu Y Zhou H Yang L. The Chinese version of hospital anxiety and depression scale: psychometric properties in Chinese cancer patients and their family caregivers. Eur J Oncol Nurs. (2016) 25:16–23. 10.1016/j.ejon.2016.09.004 27865248

[47] Buysse DJ Reynolds CF Monk TH Berman SR Kupfer DJ. The Pittsburgh sleep quality index: a new instrument for psychiatric practice and research. Psychiatry Res. (1989) 28:193–213. 10.1016/0165-1781(89)90047-4 2748771

[48] Tsai PS Wang SY Wang MY Su CT Yang TT Huang CJ. Psychometric evaluation of the Chinese version of the Pittsburgh Sleep Quality Index (CPSQI) in primary insomnia and control subjects. Qual Life Res. (2005) 14:1943–52. 10.1007/s11136-005-4346-x 16155782

[49] Hopko DR Bourland SL Stanley MA Beck JG Novy DM Averill PM. Generalized anxiety disorder in older adults: examining the relation between clinician severity ratings and patient self-report measures. Depress Anxiety. (2000) 12:217–25. 10.1002/1520-6394(2000)12:4<217::AID-DA5>3.0.CO;2-6 11195758

[50] Dennis CL Dowswell T. Interventions (other than pharmacological, psychosocial or psychological) for treating antenatal depression. Cochrane Database Syst Rev. (2013) 7:CD006795. 10.1002/14651858.CD006795.pub3 23904069 PMC11536339

[51] Wang C Pan R Wan X Tan Y Xu L Ho CS. Immediate psychological responses and associated factors during the initial stage of the 2019 Coronavirus Disease (COVID-19) epidemic among the general population in China. Int J Environ Res Public Health. (2020) 17:1729. 10.3390/ijerph17051729 32155789 PMC7084952

[52] Gao J Zheng P Jia Y Chen H Mao Y Chen S. Mental health problems and social media exposure during COVID-19 outbreak. PLoS ONE. (2020) 15:e0231924. 10.1371/journal.pone.0231924 32298385 PMC7162477

[53] Ni MY Yang L Leung CMC Li N Yao XI Wang Y. Mental health, risk factors, and social media use during the COVID-19 epidemic and cordon sanitaire among the community and health professionals in Wuhan, China: cross-sectional survey. JMIR Ment Health. (2020) 7:e19009. 10.2196/19009 32365044 PMC7219721

[54] Patel S Henderson R Bradley L Galloway B Hunter L. Effect of visual display unit use on blink rate and tear stability. Optom Vis Sci. (1991) 68:888–92. 10.1097/00006324-199111000-00010 1766652

[55] Sheppard AL Wolffsohn JS. Digital eye strain: prevalence, measurement and amelioration. BMJ Open Ophthalmol. (2018) 3:e000146. 10.1136/bmjophth-2018-000146 29963645 PMC6020759

[56] Hussaindeen JR Gopalakrishnan A Sivaraman V Swaminathan M. Managing the myopia epidemic and digital eye strain post COVID-19 pandemic - what eye care practitioners need to know and implement? Indian J Ophthalmol. (2020) 68:1710–2. 10.4103/ijo.IJO_2147_20 32709834 PMC7640876

[57] Giannaccare G Vaccaro S Mancini A Scorcia V. Dry eye in the COVID-19 era: how the measures for controlling pandemic might harm ocular surface. Graefes Arch Clin Exp Ophthalmol. (2020) 258:2567–8. 10.1007/s00417-020-04808-3 32561978 PMC7304378

[58] Fine PG. Long-term consequences of chronic pain: mounting evidence for pain as a neurological disease and parallels with other chronic disease states. Pain Med. (2011) 12:996–1004. 10.1111/j.1526-4637.2011.01187.x 21752179

[59] Kaiser T Janssen B Schrader S Geerling G. Depressive symptoms, resilience, and personality traits in dry eye disease. Graefes Arch Clin Exp Ophthalmol. (2019) 257:591–9. 10.1007/s00417-019-04241-1 30648207

[60] Tiller JWG. Depression and anxiety. Med J Aust. (2013) 199:S28–31. 10.5694/mjao12.10628 25370281

[61] Liu Q Wangqing P Baima Y Wang S Shen Z Zhou J. Comorbid depressive and anxiety symptoms and their correlates among 93,078 multiethnic adults in southwest China. Front Public Health. (2021) 9:783687. 10.3389/fpubh.2021.783687 34970528 PMC8712466

[62] Sullivan BD Crews LA Messmer EM Foulks GN Nichols KK Baenninger P. Correlations between commonly used objective signs and symptoms for the diagnosis of dry eye disease: clinical implications. Acta Ophthalmol. (2014) 92:161–6. 10.1111/aos.12012 23279964

[63] Smith JA Albenz J Begley C Caffery B Nichols K Schaumberg D. The epidemiology of dry eye disease: report of the Epidemiology Subcommittee of the International Dry Eye WorkShop (2007). Ocul Surf. (2007) 5:93–107. 10.1016/S1542-0124(12)70082-4 17508117

[64] Denoyer A Rabut G Baudouin C. Tear film aberration dynamics and vision-related quality of life in patients with dry eye disease. Ophthalmology. (2012) 119:1811–8. 10.1016/j.ophtha.2012.03.004 22591770

[65] Kawashima M Uchino M Yokoi N Uchino Y Dogru M Komuro A. Associations between subjective happiness and dry eye disease: a new perspective from the Osaka study. PLoS ONE. (2015) 10:e0123299. 10.1371/journal.pone.0123299 25830665 PMC4382322

[66] Song C Halbreich U Han C Leonard BE Luo H. Imbalance between pro- and anti-inflammatory cytokines, and between Th1 and Th2 cytokines in depressed patients: the effect of electroacupuncture or fluoxetine treatment. Pharmacopsychiatry. (2009) 42:182–8. 10.1055/s-0029-1202263 19724980

[67] Baturone R Soto MJ Márquez M Macías I de Oca MM Medina F. Health-related quality of life in patients with primary Sjögren's syndrome: relationship with serum levels of proinflammatory cytokines. Scand J Rheumatol. (2009) 38:386–9. 10.1080/03009740902973821 19575332

[68] Hackett KL Newton JL Frith J Elliott C Lendrem D Foggo H. Impaired functional status in primary Sjögren's syndrome. Arthritis Care Res. (2012) 64:1760–4. 10.1002/acr.21738 23111856

[69] Afonso AA Monroy D Stern ME Feuer WJ Tseng SC Pflugfelder SC. Correlation of tear fluorescein clearance and Schirmer test scores with ocular irritation symptoms. Ophthalmology. (1999) 106:803–10. 10.1016/S0161-6420(99)90170-7 10201606

[70] Brunes A Augestad LB Gudmundsdottir SL. Personality, physical activity, and symptoms of anxiety and depression: the HUNT study. Soc Psychiatry Psychiatr Epidemiol. (2013) 48:745–56. 10.1007/s00127-012-0594-6 23052425

[71] Ridley M Rao G Schilbach F Patel V. Poverty, depression, and anxiety: causal evidence and mechanisms. Science. (2020) 370:eaay0214. 10.1126/science.aay0214 33303583

[72] Johnson-Lawrence V Scott JB James SA. Education, perceived discrimination and risk for depression in a southern black cohort. Aging Ment Health. (2020) 24:1872–8. 10.1080/13607863.2019.1647131 31389255 PMC7004854

[73] Chen W Tian T Wang S Xue Y Sun Z Wang S. Characteristics of carotid atherosclerosis in elderly patients with type 2 diabetes at different disease course, and the intervention by statins in very elderly patients. J Diabetes Investig. (2018) 9:389–95. 10.1111/jdi.12710 28685957 PMC5835477

[74] Fergus TA Valentiner DP. Reexamining the domain of hypochondriasis: comparing the Illness Attitudes Scale to other approaches. J Anxiety Disord. (2009) 23:760–6. 10.1016/j.janxdis.2009.02.016 19339156

[75] Fulton JJ Marcus DK Merkey T. Irrational health beliefs and health anxiety. J Clin Psychol. (2011) 67:527–38. 10.1002/jclp.20769 21484800

[76] Uchino M Schaumberg DA. Dry eye disease: impact on quality of life and vision. Curr Ophthalmol Rep. (2013) 1:51–7. 10.1007/s40135-013-0009-1 23710423 PMC3660735

[77] Warwick HM Salkovskis PM. Hypochondriasis. Behav Res Ther. (1990) 28:105–17. 10.1016/0005-7967(90)90023-C 2183757

[78] Leventhal H Phillips LA Burns E. The Common-Sense Model of Self-Regulation (CSM): a dynamic framework for understanding illness self-management. J Behav Med. (2016) 39:935–46. 10.1007/s10865-016-9782-2 27515801

[79] Fink P Ørnbøl E Christensen KS. The outcome of health anxiety in primary care. A two-year follow-up study on health care costs and self-rated health. PLOS ONE. (2010) 5:e9873. 10.1371/journal.pone.0009873 20352043 PMC2844425

[80] Hart J Björgvinsson T. Health anxiety and hypochondriasis: description and treatment issues highlighted through a case illustration. Bull Menninger Clin. (2010) 74:122–40. 10.1521/bumc.2010.74.2.122 20545492

[81] Lee YB Koh JW Hyon JY Wee WR Kim JJ Shin YJ. Sleep deprivation reduces tear secretion and impairs the tear film. Invest Ophthalmol Vis Sci. (2014) 55:3525–31. 10.1167/iovs.14-13881 24833736

[82] Piña R Ugarte G Campos M Íñigo-Portugués A Olivares E Orio P. Role of TRPM8 channels in altered cold sensitivity of corneal primary sensory neurons induced by axonal damage. J Neurosci. (2019) 39:8177–92. 10.1523/JNEUROSCI.0654-19.2019 31471469 PMC6786815

[83] Segal BM Pogatchnik B Henn L Rudser K Sivils KM. Pain severity and neuropathic pain symptoms in primary Sjögren's syndrome: a comparison study of seropositive and seronegative Sjögren's syndrome patients. Arthritis Care Res. (2013) 65:1291–8. 10.1002/acr.21956 23335582 PMC4137866

[84] Kawashima M Uchino M Yokoi N Uchino Y Dogru M Komuro A. The association of sleep quality with dry eye disease: the Osaka study. Clin Ophthalmol. (2016) 10:1015–21. 10.2147/OPTH.S99620 27330271 PMC4898440

[85] Yoo SS Hu PT Gujar N Jolesz FA Walker MP. A deficit in the ability to form new human memories without sleep. Nat Neurosci. (2007) 10:385–92. 10.1038/nn1851 17293859

[86] Gujar N Yoo SS Hu P Walker MP. Sleep deprivation amplifies reactivity of brain reward networks, biasing the appraisal of positive emotional experiences. J Neurosci. (2011) 31:4466–74. 10.1523/JNEUROSCI.3220-10.2011 21430147 PMC3086142

[87] Bagherzadeh-Azbari S Khazaie H Zarei M Spiegelhalder K Walter M Leerssen J. Neuroimaging insights into the link between depression and Insomnia: a systematic review. J Affect Disord. (2019) 258:133–43. 10.1016/j.jad.2019.07.089 31401541

[88] Murawiec S Chudek J Nieves W Almgren-Rachtan A Jedrzejczak J. Increasing the dosage of pregabalin in patients with focal epilepsy decreases the frequency of seizures and ameliorates symptoms of anxiety, depression and insomnia. Eur Rev Med Pharmacol Sci. (2020) 24:13015–24. 10.26355/eurrev_202012_24207 33378053

[89] Woon LS Leong Bin Abdullah MFI Sidi H Mansor NS Nik Jaafar NR. Depression, anxiety, and the COVID-19 pandemic: severity of symptoms and associated factors among university students after the end of the movement lockdown. PLoS ONE. (2021) 16:e0252481. 10.1371/journal.pone.0252481 34043731 PMC8158968

[90] Woon LS Sidi H Nik Jaafar NR Leong Bin Abdullah MFI. Mental health status of university healthcare workers during the COVID-19 pandemic: a post-movement lockdown assessment. Int J Environ Res Public Health. (2020) 17:9155. 10.3390/ijerph17249155 33302410 PMC7762588

